# HHV-8 and EBV Positive Lymphoproliferative Disease: A Challenging Case

**DOI:** 10.5146/tjpath.2021.01540

**Published:** 2022-05-19

**Authors:** Göksenil Bülbül, Gülen Gül, Mehmet Ali Özcan, Sermin Özkal

**Affiliations:** Department of Pathology, Dokuz Eylül University, School of Medicine, Izmir, Turkey; University of Health Sciences, Tepecik Training and Research Hospital, Izmir, Turkey; Department of Hematology, Dokuz Eylul University, School of Medicine, Izmir, Turkey

**Keywords:** Castleman disease, Germinotropic lymphoproliferative disorder, EBV, HHV-8

## Abstract

Human herpes virus-8 (HHV-8) is linked to four lymphoproliferative diseases: primary effusion lymphoma, HHV-8 positive multicentric Castleman disease (MCD), HHV-8 positive diffuse large B cell lymphoma and HHV-8 positive germinotropic lymphoproliferative disorder (GLPD). The diagnosis of HHV-8 associated lymphoproliferative diseases is quite challenging because each entity is rare and has a wide morphological spectrum. Our aim is to emphasize the overlapping histopathological features of MCD and GLPD as well as to underline the importance of clinicopathological correlation in case these two entities cannot be distinguished by pathological examination.

We present here a case of an 82-year-old male patient who was examined for weight loss and multiple lymphadenopathy. Histopathological examination of the axillary lymph node revealed lymphoid follicle structures of varying shapes and sizes, including some atrophic germinal centers. Plasmablast-like cells were notable in some of these areas. HHV-8 and Epstein Barr Virus (EBV) positivity were noted in some of these cells and in a small number of cells in the mantle zone. Based on these findings; a diagnosis of “HHV-8 and EBV positive lymphoproliferative disease” was established.

Since HHV-8 positive MCD and GLPD have similar histopathological features, it may not be possible to distinguish these two entities by histopathological examination only. At this point, the importance of clinicopathological correlation becomes more evident, especially in the determination of the treatment protocol to be applied to the patient.

## INTRODUCTION

Human herpesvirus-8 (HHV8) is a herpes virus that infects the endothelium, lymphocytes, keratinocytes and bone marrow stromal cells. It is associated with four lymphoproliferative diseases: primary effusion lymphoma, HHV-8 positive multicentric Castleman disease (MCD), HHV-8 positive diffuse large B cell lymphoma and HHV-8 positive germinotropic lymphoproliferative disorder (GLPD) (1,2).

The Epstein Barr Virus (EBV) is also a lymphotropic virus from the herpesvirus family like HHV8 (3). Although both viruses are associated with various lymphoid diseases, HHV8 + / EBV + lymphoproliferation is a rare entity (4).

Because of its rarity, we present a case co-infected with HHV8 and EBV resulting in a differential diagnosis difficulty due to the similar histopathological features of HHV-8 associated lymphoproliferative diseases.

## CASE REPORT

Here we report a case of an 82-year-old male from İzmir/Turkey diagnosed with schizophrenia, Parkinson’s disease and diabetes mellitus and who had been taking medications for many years. He presented to a physician with increasing weight loss for the last one year in addition to fatigue. It was also learned that his brother had a diagnosis of lymphoma. Physical examination revealed conglomerated and fixed multiple lymphadenopathies, the largest of which was 4 cm in the right inguinal region and 1 cm in the left supraclavicular region. Peripheral blood test revealed the following: hemoglobin 9.9 g/dL, white blood cells 13,200/mm³, and platelets 525,000/ mm³. There were abnormal findings in routine blood tests: serum electrolytes were generally low, BUN was 33 mg/dL, and CRP 58.6 mg/L; the IgG level was 3059.8 mg/dL (N: 700-1600) and the IgA level 502.9 mg/dL (N: 70-400). On serological examination, there was no evidence of HIV infection.

Abdominopelvic ultrasound showed hepatosplenomegaly while PET revealed cervical, supraclavicular, axillary, mediastinal-hilar, intraabdominal, bilateral inguinal and femoral multifocal lymphadenopathy in addition to bilateral pleural effusion. The largest lymph node was in the right axillary with a size of 30x18 mm and SUVmax of 3.3.

**Figure 1:**
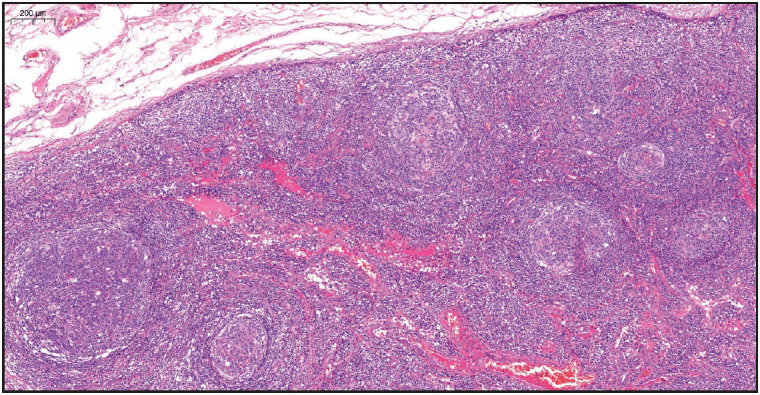
Microscopic examination of lymph node, lymphoid follicle structures of varying shapes and sizes (H&E stain, x4).

**Figure 2:**
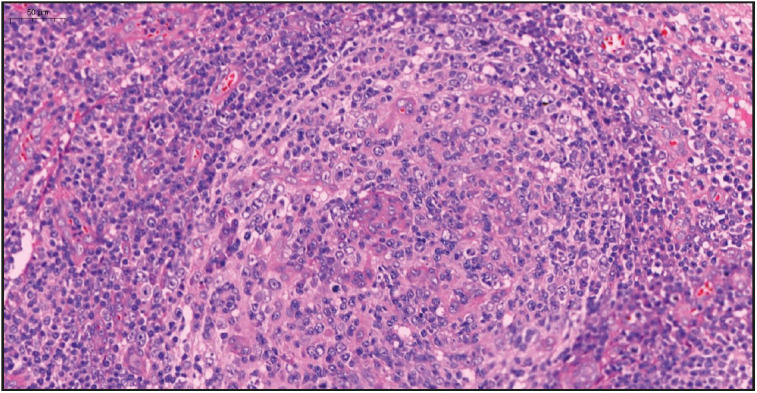
Hyalinized germinal center including plasmablast-like cells (H&E stain, x20).

**Figure 3:**
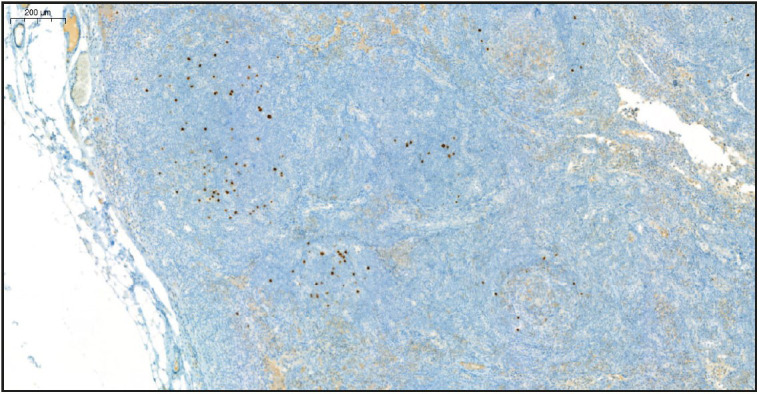
HHV-8 positive cells in the germinal center and mantle zone (HHV-8 Immunohistochemistry, x5).

**Figure 4:**
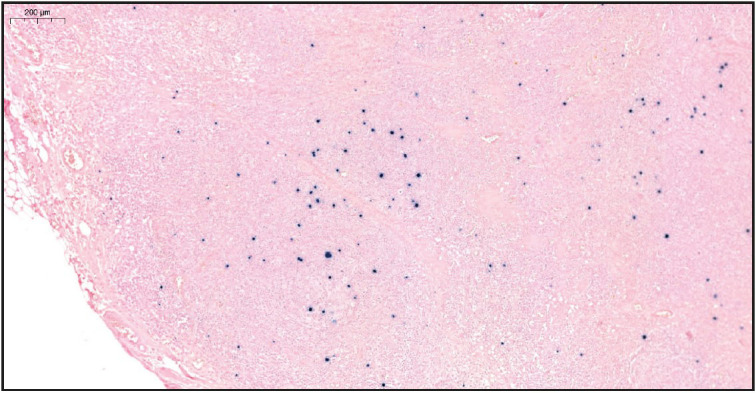
EBV positive cells in the germinal center and mantle zone by in situ hybridization (CISH EBER, x5).

**Figure 5:**
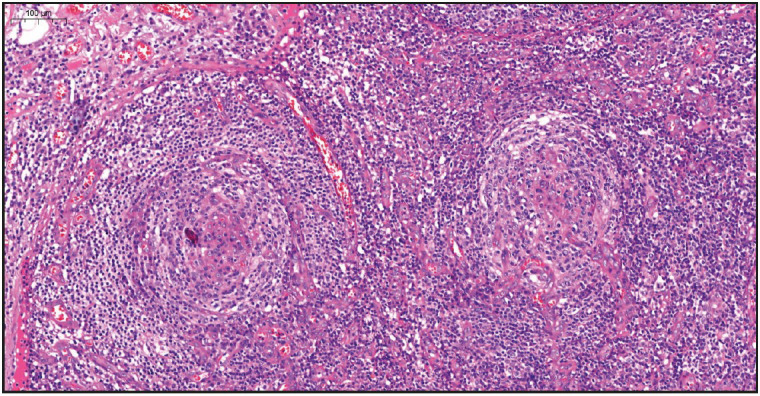
Concentric arrangement in the mantle zone areas and vascular structure penetrating into the germinal center (H&E stain, x10).

**Figure 6:**
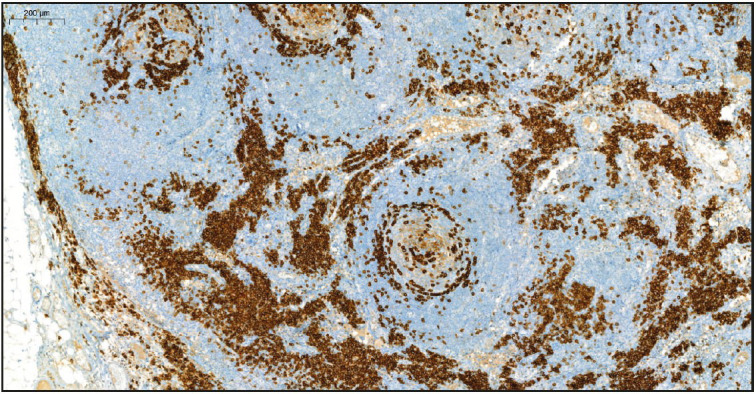
CD38 positive plasma cells forming large aggregates in interfollicular areas (CD38 Immunohistochemistry, x5).

On evaluation of the resected right axillary lymph node specimen measuring 23x13x7 mm, serial sections were gray-white colored and a nodular appearance was remarkable. In the sections of the total processed lymph node, the normal structure was partially preserved and lymphoid follicle structures (CD20 and PAX5 positive) of varying shapes and sizes, including some atrophic germinal centers (CD21 and CD23 positive, Bcl-2 negative) were observed (Figure 1). In some of the germinal central structures, it was seen that lymphoid cells were decreased and hyalinized. Plasmablast-like cells were notable in some of these areas (Figure 2). HHV-8 and EBV positivity was noted by in situ hybridization (EBER) in some of these cells and in a small number of cells in the mantle zone (Figure 3, Figure 4). In some follicle structures, a concentric arrangement in the mantle zone areas and vascular structure penetrating into the germinal center were noteworthy (Figure 5). Occasionally, interfollicular areas were enlarged. In these areas, mostly CD3 positive T lymphocytes as well as CD38 positive plasma cells, some of which formed large aggregates, and marked vascular proliferation in the endothelium were seen (Figure 6). Although plasma cells and plasmablast-like cells were predominantly lambda positive, some of them were positive with lambda and some with kappa.

Finally the case was reported as “HHV-8 and EBV Positive Lymphoproliferative Disease” instead of giving a definite diagnosis. Two cycles of Rituximab one month apart were administered to the patient.

## DISCUSSION

Kaposi sarcoma associated herpesvirus (KSHV), also known as HHV-8, is a lymphotropic virus and associated with 4 lymphoproliferative diseases: primary effusion lymphoma (PEL), HHV-8 positive multicentric Castleman disease (MCD), HHV-8-positive diffuse large B-cell lymphoma and rarely germinotropic lymphoproliferative disorder (GLPD) (2).

PEL presents as serous effusion in body cavities (peritoneal, pleural and pericardial) or solid tumour without effusion (“solid” PEL) and occurs in immunodeficient patients with HIV infection. Infected patients have systemic symptoms and prognosis is poor (5). That patients have HHV-8 and EBV positive immunoblasts with plasmacytoid cytoplasm and pleomorphic nuclei. PEL differs from GLPD in the absence of cytoplasmic immunoglobulin expression (6).

Castleman disease (CD) describes 4 diseases: unicentric CD, HHV-8 associated MCD, POEMS associated MCD, idiopathic -who are negative for HHV-8 and HIV-MCD (7,8). MCD is characterized by enlarged lymph nodes in multiple regions and spleen involvement. It is a systemic disease and involves hepatomegaly, splenomegaly, constitutional symptoms and cytopenias (9). HHV-8 associated MCD occurs in most commonly HIV positive patients but HIV negative patients have also been reported (10). Histopathology is prominent, includes hyperplastic/atrophic germinal centers and hypervascularization; plasmablasts generally located in mantle zones (11,12).

GLPD is a rare HHV-8 associated lymphoproliferative disorder, first described in 3 cases in 2002 by Du et al. and followed by 15 more case reports (12) (Table 1). It presents as localized lymphadenopathy and on histopathological examination it is characterized by an inﬁltration of germinal centers by plasmablastic cells, which are coinfected by HHV-8 and EBV. Migration of neoplastic B-lymphocytes into germinal centers may be the origin of plasmablasts in GLPD. The presence of the atypical plasma cells in the mantle zone and interfollicular area supports this theory. In addition to plasmablastic cells, residual follicle centers can be seen. There are sometimes atrophic follicles similar to MCD. GLPD responds well to chemotherapy and radiotherapy.

**Table 1:** Clinicopathological features of patients diagnosed with germinotropic lymphoproliferative disorder

	**Age/Sex**	**Clinical** **Presentation**	**HIV**	**Ig heavy/light chain expression**	**Treatment and Prognosis**
**Case 1 **(12)	41y/M	Axillary and cervical lymph node enlargement for 6 years	-	Lambda cIgM, cIgD	CHOP Complete remission
**Case 2 **(12)	61y/M	Submandibular and inguinal lymph node enlargement for 4 years Slightly enlarged spleen	-	Lambda cIgA	Excision and radiotherapy Complete remission
**Case 3 **(12)	63y/F	Paresthesia Left leg swelling Paraaortic lymph node enlargement	NI	Kappa	NI
**Case 4 **(12)	60y/M	Localized cervical lymphadenopathy	-	Kappa cIgM	Excision No evidence of relaps
**Case 5 **(13)	65y/M	Right cervical lymph node enlargement	-	Kappa cIgM	Without therapy, alive 7 years
**Case 6 **(14)	75y/M	Mass in the neck Cervical lymph node enlargement Cystic lymph node in left submandibular area	-	Kappa	CHOP 19 months disease free
**Case 7 **(15)	49y/F	Right jugulo-cervical nodal mass	-	Lambda	Excision and radiotherapy Complete remission
**Case 8 **(11)	84y/F	Multifocal lymphadenopathy	-	None	CHOP Complete remission
**Case 9 **(11)	58y/M	Localized right axillary mass for 10 years Mild splenomegaly	+	None	Resection One year later developed DLBCL, died due to his disease subsequent
**Case 10 **(16)	72y/F	Palpable left cervical lymph node	-	Lambda	Without therapy No evidence of relaps
**Case 11 **(1)	63y/F	Autoimmune hemolytic anemia Prominent mesenteric lymphadenopathy	-	Lambda	Without therapy 8 months later HHV8 + EBV + lymphoma
**Case 12 **(17)	53y/M	Swelling of cervical nodes	-	μ	NI
**Case 13 **(18)	86y/M	Localized cervical lymphadenopathy	-	Kappa	Without therapy No evidence of relapse
**Case 14 **(18)	52y/M	Inguinal lymph node enlargement for 3 years	-	None	CHOP
**Case 15 **(18)	47y/M	Generalized lymphadenopathy B Symptoms	+	None	CHOP
**Case 16 **(18)	27y/M	Generalized lymphadenopathy B Symptoms	+	Kappa	Rituximab
**Case 17 **(18)	30y/M	Generalized lymphadenopathy B Symptoms	+	Kappa	R-DA-EPOCH
**Case 18 **(18)	42y/M	Generalized lymphadenopathy B Symptoms	+	Lambda	R-DA-EPOCH

**NI:** No information, **DLBCL:** Diffuse Large B Cell Lymphoma, **CHOP:** Rituximab, cyclophosphamide, doxorubicin, vincristine, prednisone, **EPOCH:** Etoposide, prednisone, vincristine, cyclophosphamide, doxorubicin, **R-DA-EPOCH:** Rituximab, vincristine, adriamycin, cyclophosphamide, methylprednisolone.

In keeping with these features, the possibilities of “HHV-8 Positive Multicentric Castleman Disease” and “HHV-8 Positive Germinotropic Lymphoproliferative Disorder” were considered in the differential diagnosis of our case. Although MCD and GLPD are two distinct diseases, similar/overlapping histopathological features can be seen in these two entities (Table 2).

**Table 2:** Comparison of the clinical and pathological features of HHV-8 positive MCD and GLPD.

	**HHV-8 Positive MCD**	**HHV-8 Positive GLPD**
**Clinical Presentation**	Mostly in HIV positive immunodeficient patients Generalized lymphadenopathy, splenomegaly, constitutional symptoms	Predominantly in HIV negative immunocompetent patients Often localized lymphadenopathy Sometimes multifocal lymph node involvement and rarely systemic symptoms
**Prognosis**	Poor prognosis	Usually favorable response to chemotherapy and radiotherapy
**Microscopic Findings**	Abnormal follicle structures Plasmablasts generally located in the mantle zone but they may intrude into germinal centers Atrophic or hyperplastic germinal centers Prominent vascular proliferation Concentric onion skin-like layering Plasma cell hyperplasia in interfollicular area	Residual follicle centers can be seen Plasmablasts partially/completely invade germinal centers Sometimes atrophic follicles similar to MCD
**EBER**	Positive/Negative	Always positive
**HIV**	Usually positive, rarely negative	Predominantly negative, rarely positive
**Cytoplasmic Ig Heavy Chain**	Elevated, only IgM	Elevated, any heavy chain
**Ig Light Chain**	Monotypic lambda +	Monotypic kappa or lambda +
**Clonality (Ig gene rearrangements)**	Polyclonal	Polyclonal/Oligoclonal
**Mutated Ig Genes**	Absent	Present
**Cell of Origin**	A naive B cell	A germinal center B cell

As MCD progresses with systemic involvement, the multiple lymph node involvement and systemic symptoms in our patient primarily directed us to a diagnosis of MCD. Although GLPD usually presents as localized and sometimes multifocal lymphadenopathy (12), a few cases with symptoms such as mild splenomegaly and systemic symptoms have been reported (10,11).

Since GLPD is mostly seen in HIV-negative immunocompetent patients, we may consider the HIV negativity in favor of GLPD in our patient. However there is also a 58-year-old HIV-positive patient who was diagnosed with GLPD in the literature (11). In addition, an HIV-negative HHV-8 positive subgroup of MCD, which occurs mostly in immunosuppressive patients, has also been identified (10). Therefore, the HIV status of the patient is not a reliable criterion in distinguishing these two diseases.

Some features described in microscopic findings (plasmablast-like cells, atrophic germinal centers, decreased lymphoid cells, hyalinization etc.) overlap with both entities but the presence of a concentric arrangement in the mantle zone strengthens the diagnosis of MCD.

Another important point according to all published GLPD cases in the literature is that HHV-8 and EBV co-infection is one of the most significant criteria that differentiates GLPD from MCD (11–13,15,18,19). However, in an article published by Nobel et al. in 2019, EBV positivity was detected in two of two HHV-8 positive MCD patients included in the study (20). This newly defined condition, the presence of EBV positivity in MCD, will cause serious difficulties in distinguishing these two diseases, as in our case (21,22).

Due to the reasons described above and the morphologically similar features, it is very difficult to distinguish between the two entities only by histopathological examination. At this point, the importance of clinicopathological correlation becomes more evident, especially in the determination of the treatment protocol applied to the patient. The physical examination and laboratory findings should also be evaluated in detail and carefully.

## Conflict of Interest

The authors declare no conflict of interest.
